# Transcriptomic Analysis Reveals the Roles of Detoxification Systems in Response to Mercury in *Chromera velia*

**DOI:** 10.3390/biom9110647

**Published:** 2019-10-24

**Authors:** Abdoallah Sharaf, Roberto De Michele, Ayush Sharma, Safieh Fakhari, Miroslav Oborník

**Affiliations:** 1 Institute of Parasitology, Biology Centre, Czech Academy of Sciences, 37005 České Budějovice, Czech Republic; ayush.sharma@paru.cas.cz; 2 Genetic Department, Faculty of Agriculture, Ain Shams University, Cairo 11241, Egypt; 3 Institute of Biosciences and Bioresources (IBBR), National Research Council (CNR) of Italy, 90129 Palermo, Italy; roberto.demichele@cnr.it (R.D.M.); safiehfakhari@gmail.com (S.F.); 4 Faculty of Science, University of South Bohemia, 37005 České Budějovice, Czech Republic

**Keywords:** chromerids, transcriptome, heavy metal, antioxidant enzymes, xenobiotics, phylogenies, reactive oxygen species, reactive nitrogen species

## Abstract

Heavy metal pollution is an increasing global concern. Among heavy metals, mercury (Hg) is especially dangerous because of its massive release into the environment and high toxicity, especially for aquatic organisms. The molecular response mechanisms of algae to Hg exposure are mostly unknown. Here, we combine physiological, biochemical, and transcriptomic analysis to provide, for the first time, a comprehensive view on the pathways activated in *Chromera velia* in response to toxic levels of Hg. Production of hydrogen peroxide and superoxide anion, two reactive oxygen species (ROS), showed opposite patterns in response to Hg^2+^ while reactive nitrogen species (RNS) levels did not change. A deep RNA sequencing analysis generated a total of 307,738,790 high-quality reads assembled in 122,874 transcripts, representing 89,853 unigenes successfully annotated in databases. Detailed analysis of the differently expressed genes corroborates the biochemical results observed in ROS production and suggests novel putative molecular mechanisms in the algal response to Hg^2+^. Moreover, we indicated that important transcription factor (TF) families associated with stress responses differentially expressed in *C. velia* cultures under Hg stress. Our study presents the first in-depth transcriptomic analysis of *C. velia*, focusing on the expression of genes involved in different detoxification defense systems in response to heavy metal stress.

## 1. Introduction

Pollution with heavy metals is a major environmental concern worldwide because of their diffusion, persistence, and toxic effects. Heavy metals are released in the ecosystems during volcanic eruptions and by anthropogenic activities, such as mining, fossil fuel burning, agriculture, and waste management. Heavy metals are not degraded by organisms, but can be absorbed by plants and algae, thus entering the food chain. Among heavy metals, mercury (Hg) is particularly toxic, since it can accumulate at a high level, especially in fishes, and cause serious diseases, such as the infamous Minamata outbreak in Japan in the 1950s [1]. In the environment, Hg is present in different forms, i.e., as elemental Hg, ionic Hg^2+^, or generated as methyl-Hg. Regardless of the form, a large part of Hg ends up in the oceans, with estimates of about 290 million moles currently released [2]. Hg quickly accumulates in both terrestrial plants [3,4,5] and aquatic organisms [6,7,8,9]. In oceans, algae are the first target of Hg accumulation, and some microalgal species are active Hg-accumulators and may serve for phytoremediation purposes [10].

Physiological effects of heavy metals on higher plants, especially cadmium, have been extensively studied (recently reviewed by [11]). Far less is known about the plant physiological responses to Hg. In plants, high concentrations of mercury are usually very toxic and lead to cell death, often preceded by water loss, impaired ion homeostasis, and disruption of membrane integrity (reviewed by [12,13]). Events often associated with exposure to heavy metals are the oxidative and nitrosative bursts.

Various metabolic processes generate reactive oxygen and nitrogen species (ROS and RNS) as by-products [14,15] or in response to biotic and abiotic stresses [16]. ROS include singlet oxygen (^1^O_2_), superoxide anion (O^2−^), hydrogen peroxide (H_2_O_2_), and hydroxyl radical (·OH) [17]. RNS comprises, among others, nitric oxide (NO) and peroxynitrite (ONOO^−^). ROS and RNS are reactive molecules that can directly damage proteins, lipids, and DNA, but they also act as signaling molecules during biotic and abiotic stress responses [15,18]. In physiological conditions, ROS homeostasis is maintained by the antioxidant machinery of the cell, including the enzymes superoxide dismutase (SOD), catalase (CAT), glutathione peroxidase (GPX), ascorbate peroxidase (APX), peroxidase (POD), and glutathione reductase (GR); and non-enzymatic mechanism such as glutathione (GSH), ascorbate (AsA), and others [14,18,19].

It is well known that ROS and RNS play important roles during plant response to heavy metals, and that, often, different reactive species interact. For instance, *Arabidopsis* cells treated with cadmium trigger an early wave in NO, required for a later H_2_O_2_ burst and cell death [20]. In the case of Hg, the little information available is restricted to H_2_O_2_ and O^2−^, and it shows some contradictory patterns, with their levels increasing in some species/tissues, and decreasing in others [21,22,23,24].

An involvement of ROS in Hg-induced toxicity is also suggested by the evidence that the antioxidant machinery is altered following treatment with Hg^2+^, although once again the modulation of each enzyme activity varies greatly depending on the experimental system, species, duration, and intensity of treatments [4,8,21,23,24,25,26,27].

Cells can detoxify from heavy metals by transforming them into less toxic forms. Photosynthetic organisms perform biotransformation in three phases [28,29,30,31,32]. During phase I (transformation), oxidations, reductions, or hydrolysis are catalyzed by mixed-function oxidases (MFO) (i.e., cytochrome P-450 (Cyt P450), cytochrome b5 (Cyt b5), and NADPH-cytochrome P450 reductase (P450R) [33,34,35]. The MFO system can transform lipophilic xenobiotics, such as methyl-Hg, into more water-soluble compounds to facilitate their excretion [36]. Phase II of biotransformation (conjugation) facilitates the heavy metal excretion through binding with chelating compounds such as GSH and glucuronic acid (GA) [37,38]. The biotransformation detoxication pathway is acting as a sink for environmental chemicals in microalgae and could function as ‘‘green livers’’ since it is similar to those of the mammalian liver [30,34,39] Phase III involves confining the heavy metal in the cell wall or the vacuole [40,41,42]. In animals, multixenobiotic resistance (MXR) P-glycoprotein transporter (ABC) can also export xenobiotic compounds [43,44]. Very little is known about the biotransformation pathways in algae.

The heavy metal response also involves an active transcriptional rearrangement. Several transcription factors (TFs) have been identified to play a role in heavy metal stress response, acting upstream of ROS signaling [45]. Under heavy metal stress, the activities of both POD and SOD increased due to the overexpression of *ThbZIP*1 in tobacco [46]. *WRKY* transcription factors provide defense against various stresses, including heavy metals [47]. ZmWRKY4 was reported to regulate the expression of *SOD* and *APX* in response to Cd stress in maize [48].

*Chromera velia* is a unicellular alveolate alga living in an association with stony corals in Australia [49,50]. Together with the other chromerid *Vitrella brassicaformis*, *C. velia* represents the closest known phototrophic relatives to apicomplexan parasites, branching together with predatory colpodellids at the root of the parasitic Apicomplexa [51]. Chromerids possess a unique photosynthetic apparatus, which seems to be well adapted to high photosynthesis-related oxidative stress [52,53,54]. Photosystem I (PSI) of *C. velia* non-canonically contains two permanently attached SOD molecules and several protein subunits with no sequence homology to known function [55]. Such organization of photosystems could provide a framework for the efficient and adaptable photosynthesis observed in *C. velia* [52,55].

This study aims to unravel the physiological responses of the marine protist *C. velia* to toxic Hg^2+^ exposure. Due to the double role of *C. velia* as a model for the study of apicomplexan parasites and microalgae, the knowledge acquired by this study might be of interest for both environmental and medical purposes. We tackled the response with a physiological, biochemical, and transcriptomic approach, to identify the molecular mechanism of the stress response in *C. velia*. RNA-Seq is an efficient technology for transcriptomics studies [56]. In recent years, RNA-seq has proven useful for the identification of functionally related genes and their expression patterns in algal species responding to abiotic stress [57,58,59,60].

In this study we: (i) Characterize the physiological response of *C. velia* cultures to different concentrations of Hg^2+^, over time, assessing the toxicity effects and modulation of different ROS and RNS species; (ii) present the first deep transcriptome analyses for *C. velia*; (iii) identify differentially expressed genes (DEGs); (iv) investigate the molecular regulatory network of detoxification systems in *C. velia*; and (v) identify TFs involved in response to Hg exposure. This work provides a basic knowledge of the mechanisms used by *Chromera* to cope with heavy metal toxicity.

## 2. Materials and Methods

### 2.1. Chromera velia Culture and Treatment

*C. velia* was cultivated in F2 medium [49] made with fresh seawater collected in Sicily, Italy, filtered through Miracloth and pasteurized at 80 °C for 7 h. Minerals and vitamins were added at double concentration, compared to the original recipe. Cells were kept at 25 °C in polypropylene flasks under artificial light (30–50 μmol PAR/m2/s) with a day/night photoperiod 16 h/8 h. Subculturing was scheduled every two weeks, when cells were in the mid-exponential growth phase (Supplementary Figure S1) at approximately 6.5 × 10^7^ cells/mL, by diluting 30 mL of inoculum in 300 mL fresh medium.

For ROS, RNS, cell viability, and chlorophyll measurements, cells were treated 10 days after subculturing, by adding 0, 0.3, 0.9, and 3 mL of a 10 mM HgCl_2_ stock solution to 300 mL cultures, to yield 0, 10, 30, and 100 µM final concentrations, respectively. Measurements were performed 3, 6, 24, 48, and 72 h after treatment. Depending on the experiment, we performed three or four experimental rounds. In each experimental round, each treatment was run in three independent replicates. *t*-test was performed on all the analyses, and significant differences were scored for *p* < 0.05.

For the transcriptomic analysis, 300 mL of 10 day-old cultures were treated with either 0.9 mL HgCl_2_ (30 µM final concentration) or an equal amount of pure water (mock) for 6 h. Cells were then harvested by filtration, weighted, and immediately frozen in liquid nitrogen until RNA extraction. The experiment was run in triplicate (three treated samples, three mock-controls).

### 2.2. Cell Count and Chlorophyll Quantification

To measure cell growth, we counted the cells present in the culture at a binocular microscope (200×) with the aid of a Burker slide chamber. Values are reported as the mean of 12 squares.

For chlorophyll quantification, we measured chlorophyll a, the only chlorophyll present in *C. velia* [54]. We followed the protocol described in [61], with some modifications. Briefly, 10 mL culture was centrifuged at 2000 rcf for 2 min to collect the cells in the bottom of a plastic tube. Cells were suspended in 1.5 mL of absolute ethanol in a plastic microtube and ground using a mortar and pestle with 100 mg of sand. Chlorophyll was then extracted by incubating the samples at 60 °C for 30 min in agitation (150 rpm). Quantification was performed in the Synergy H1 reader (Biotek, Winooski, VT, USA) by reading absorbance at 649 and 645 nm, and by applying the formula: Chlorophyll a (µg/mL) = 13.7 A_665_ − 5.76 A_649_ [62].

### 2.3. ROS and RNS Quantification

ROS and RNS were quantified by fluorimetric analysis using specific fluorescent dyes. In particular, 2,7-dichlorodihydrofluorescein diacetate (H_2_DCF-DA) is an intracellular marker that becomes fluorescent when oxidized [63]; dihydroethidium (DHE) is a specific marker for the superoxide anion (·O^2−^) [64]; 4-Amino-5-methylamino-2′,7′-difluorofluorescein diacetate (DAF-FM-DA) is an intracellular specific marker for nitric oxide (NO) [65]; Aminophenyl fluorescein (APF) is a marker for peroxynitrite (ONOO^−^) and hydroxyl radical (⋅OH), since the dye is unable to discriminate between the two molecules [66]. Dyes were dissolved in DMSO to a stock concentration of 10 mM (H_2_DCF-DA, DHE, APF) or 5 mM (DAF-FM-DA). All dyes were from Cayman Chemicals, USA. Two mL of culture was deposited in a well in a transparent 12-well polypropylene plate (Greiner Frickenhausen, Germany) and brought to pH 7.5 by adding 20 µL 10 mM Tris buffer (final concentration 100 µM). Then, 2 µL of dye was added to the culture, with final dye concentration of 10 µM (H_2_DCF-DA, DHE, APF) or 5 µM (DAF-FM-DA). Plates were incubated, in the dark, at 25 °C for 30 min on agitation (100 rpm). Fluorescence was then measured at a Synergy H1 reader (Biotek, USA) with a bottom reader mode and gain set to 80 (H_2_DCF-DA and APF) or 100 (DHE and DAF-FM-DA) and bandwidth of 9 nm. Due to the possible formation of cell clumps, which could affect the homogeneity of the fluorescence readout, the measurement was made in 21 different points on the well surface and averaged (“area scan” mode). Excitation and emission wavelengths for each dye were 495/525 Ex/Em for H_2_DCF-DA; 495/515 Ex/Em for DAF-FM-DA and APF; for DHE, we used 405/570 nm Ex/Em instead of the commonly used 480/580 nm Ex/Em because the former setup was proved to be more selective in detecting O^2−^, rather than the unspecific oxidized byproduct 2-OH-ethidium [64].

For extracellular H_2_O_2_ quantification, we used the xylenol orange protocol described in [67], with some modifications. Briefly, 2 mL of culture were filtered through a chromatographic column (Poly-Prep; Bio-Rad, Hercules, CA, USA) to separate cells from the growth medium. An aliquot of 500 µL of the flow through was added to an equal volume of assay reagent (500 mM ferrous ammonium sulfate, 50 mM H_2_SO_4_, 200 mM xylenol orange, and 200 mM sorbitol) and incubated for 45 min in the dark. The H_2_O_2_-mediated oxidation of Fe^2+^ to Fe^3+^ was determined by measuring the A_560_ of the Fe^3+^-xylenol orange complex. All reactions were carried out at least in duplicate, and their reproducibility was checked.

### 2.4. RNA Isolation, cDNA Library Construction, and Sequencing

TRI Reagent (Sigma-Aldrich, St. Louis, MO, USA) was used to extract total RNA from the six individual samples. Approximately 1.5 μg of the extracted RNA was used for complementary DNA (cDNA) library construction and subsequent Illumina sequencing. The six cDNA libraries (Cvel_cont1, Cvel_cont2, Cvel_cont3, Cvel_mer1, Cvel_mer2, and Cvel_mer3) were generated using NEBNext UltraTM RNA Library Prep Kit for Illumina (NEB, USA) following the manufacturer’s instructions [68]: (i) Poly-T oligo-attached magnetic beads were used to purify mRNA; (ii) fragmentation was carried out under high temperature in NEBNext; (iii) M-MuLV Reverse Transcriptase (RNase H −) was used to synthesize first strand cDNA using random hexamer primer.; (iv) the cDNA fragments were isolated and purified with AMPure XP system (Beckman Coulter, Beverly, USA) after synthesis second cDNA strand and adaptor ligation; (v) then, the final cDNA library was created based on PCR amplification, and library quality was assessed on the Agilent Bioanalyzer 2100 system (Santa Clara, CA, USA). The resulting per cDNA library was sequenced using the Illumina NextSeq 550 platform (San Diego, CA, USA) following the manufacturer’s recommendations at the Central European Institute of Technology of Masaryk University (Brno, Czech Republic).

### 2.5. Transcriptome Analysis

The RNA-Seq reads were assembled as previously described [68,69]. The assembled unigenes were annotated by BLAST search against protein databases, including NCBI non-redundant protein, Protein family (Pfam), Swiss-Prot, Protein sequence analysis and classification (InterPro), Kyoto Encyclopedia of Genes and Genomes (KEGG), and Gene Ontology (GO) with an E-value cutoff of 10^−5^. The best-aligning results from Nr, InterPro, and Swiss-Prot databases were selected as coding region sequences of unigenes. If the results from two databases conflicted with each other, a priority order of Nr and Swiss-Prot was considered. Meanwhile, Trinotate was used to archive all functional annotation data derived from the analysis of transcripts and integrate them into an SQLite database [70]. Also, the GO functional annotation of unigenes was gained using an in-house script, and GO functional classification was obtained to classify the possible functions of the unigenes based on Nr annotations using REViGO [71].

### 2.6. Quantification of Gene Expression Levels

RSEM software package was used to estimate the gene expression levels [72] for each sample: (i) The clean reads mapped to the de novo reference assembled transcriptome; (ii) the reads count was obtained for each gene; (iii) the gene expression level was normalized to FPKM (fragments per kilobase of transcript per millions fragments) based on the number of reads count. Differential expression analysis of the control and Hg-treated samples was performed using the edgaR R package (3.24.3) [73]. The threshold for differential expression was adjusted to *p*-value < 10^−3^ using the Benjamini–Hochberg method.

### 2.7. Molecular Phylogeny

Our assembled unigene was searched for selected detoxification-related enzymes (heme oxygenase (HO), superoxide dismutase (SOD), NADPH-cytochrome P450 reductase (P450R), Cytochrome P450 (CYP), and Catalase-peroxidase (katG)) by deltaBLAST [74]; also, the genome of *C. velia* [75] was searched through the Cryptosporidium Genomics database (CryptoDB) [76] using previously characterized amino acid sequences from eukaryotes, bacteria, and archaea as queries. To assess support for our chosen gene models, we checked whether the 5′-end of the gene model was transcribed by performing BLAST against the identified RNA-Seq contigs in *C. velia* CCMP2878 transcriptome in normal culture conditions RNA-Seq datasets [75]. We predicted all potential N-termini and targeting tags of *C. velia* and its close relative species *V. brassicaformis* detoxification-related enzymes: (Heme oxygenase (HO); superoxide dismutase (SOD); NADPH-cytochrome P450 reductase (P450R); Cytochrome P450 (CYP); and Catalase-peroxidase (katG)) by SignalP 3.0 [77], SignalP 4.1 [78], ASAFind [79], TargetP 1.1 [80], iPSORT [81], WoLF PSORT [82], Mitoprot [83], and the eukaryotic protein subcellular localization predictor (DeepLoc-1.0) [84] (Supplementary Table S1).

Amino acid sequences of all five detoxification-related enzymes: (Heme oxygenase (HO); Superoxide dismutase (SOD); NADPH-cytochrome P450 reductase (P450R); Cytochrome P450 (CYP); and Catalase-peroxidase (katG)) from *V. brassicaformis* were retrieved from the Cryptosporidium Genomics database [76], whereas plants, green and red algae, alveolates (apicomplexans and ciliates), stramenopiles (oomycetes and diatom), metazoans, fungi, kinetoplastids, amoebozoans, parabasalids, and diplomonads sequences were downloaded from NCBI GeneBank and specialized databases. We also considered the available bacteria and viruses amino acid sequences in NCBI GeneBank, with particular emphasis placed on those prokaryotes that could serve as a donor of genes transferred during endosymbiotic events, such as cyanobacteria (sharing an ancestor with chloroplasts) and α-proteobacteria (sharing a common ancestor with mitochondria). Sequences were aligned using MAFFT version 7 software [85]. Ambiguously aligned sequences and gaps were excluded for further analysis. Alignments were tested using Prottest [86] to choose the appropriate model for amino acid substitution. Maximum likelihood method using RAxML [87] and IQ-TREE v1.6.10 [88] were used to construct phylogenetic trees.

### 2.8. RT-qPCR Analysis

The quantitative reverse transcription PCR (RT-qPCR) analysis was performed using three biological and three technical replicates of total RNA. First strand cDNA fragments were synthesized using the SuperScript IV RT (Invitrogen, Carlsbad, CA, USA). RT-qPCR was performed on the LightCycler 480 system (F. Hoffmann-La Roche Ltd., Basel, Switzerland) using a TP SYBR 2× Master Mix (Top-Bio, Prague, Czech Republic) in 25 μl reaction mixtures. The PCR reaction protocol was 94 °C for 10 min, 55 cycles of 94 °C for 10 s, 60 °C for 10 s, and 72 °C for 10 s. The fluorescence was measured via a 65–95 °C melting curve. The specific primers for RT-qPCR were designed using Geneious prime software [89] (Supplementary Table S2A). The relative expression level of the selected genes using the Actin gene as the internal control gene was calculated using ratio = 2 ^−ΔΔCT^.

### 2.9. Data Deposition

The six datasets of RNA-sequencing are available at the NCBI Short Read Archive (SRA) with the GenBank accession No.: PRJNA529782. The database archives all functional annotation data derived from the analysis was deposited into Mendeley Data with DOI: 10.17632/pkn2f6dzt3.1.

## 3. Results and Discussions

### 3.1. Mercury Toxicity

**Figure 1:**
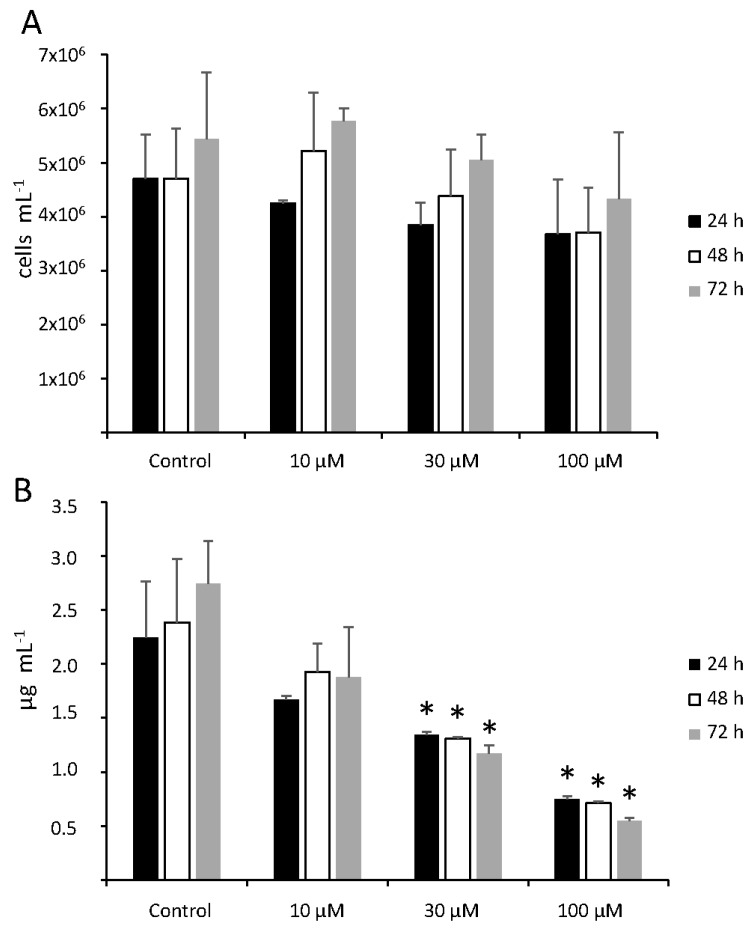
Cell number (**A**) and chlorophyll a content (**B**) of cultures treated with 0 (control), 10, 30, and 100 µM HgCl_2_ for 3 h, 6 h, 24 h, 48 h, and 72 h. *N* = 3 ± Standard Deviation (SD). Asterisks indicate significant differences compared to the corresponding control value, for *p* < 0.05.

In conditions of nutrient starvation, *C. velia*, like many other microorganisms, enters in a dormant state, and it is less susceptible to environmental triggers [50]. Since the aim of this study was to study the physiological responses to Hg^2+^, we first characterized the growth curve of *C. velia* culture in our experimental conditions, to determine the best time for treatments. After subculturing, the culture experienced about three days of the initial lag phase (Supplementary Figure S1). By approximately day four, it entered into the exponential growth phase, that lasted for about two weeks. After that, if not subcultured, nutrients were depleted, and the culture rested in a stationary phase. In our previous experience with plant cell cultures and cadmium, we observed that treatments in the 50–150 µM range were able to trigger physiological responses and eventually cell death [20]. Therefore, for initial screening, we selected similar range of HgCl_2_ concentrations (10, 30, 100 µM) and times of treatment (from 3 to 72 h) to study the physiological response of *C. velia* under different acute stress conditions, and to select the best condition for subsequent transcriptomic analysis. First, we measured cell growth and chlorophyll content, as indicators of Hg toxicity. Treatment with 10 µM Hg^2+^ did not affect cell growth (Figure 1A). At 30 µM and 100 µM treatment, we observed a dose-dependent reduction in cell number, although not statistically significant with *p* > 0.5 in all cases. The modest effect in cell growth was not surprising, considering the slow growth of *C. velia* cultures during the three days of treatment (Supplementary Figure S1, Figure 1A control).

Conversely, chlorophyll content showed a marked reduction when cultures were treated with Hg^2+^, in a dose-dependent fashion (Figure 1B). In untreated cultures, chlorophyll content increased slightly over time, a result in agreement with the higher number of cells counted in the same time frame. In cultures treated with 30 and 100 µM Hg^2+^, chlorophyll content was sharply reduced in all time points, indicating a toxic effect of Hg^2+^ at these concentrations and time points of treatment. A similar effect of high Hg^2+^ concentrations on chlorophyll content had been observed on the microalgae *Coccomyxa subellipsoidea* [26] and *Chlamydomonas reinhardtii* [8,90], and on cucumber cotyledons [91], confirming that Hg^2+^ is toxic for plants and algae at high concentrations.

### 3.2. ROS and RNS Response

**Figure 2:**
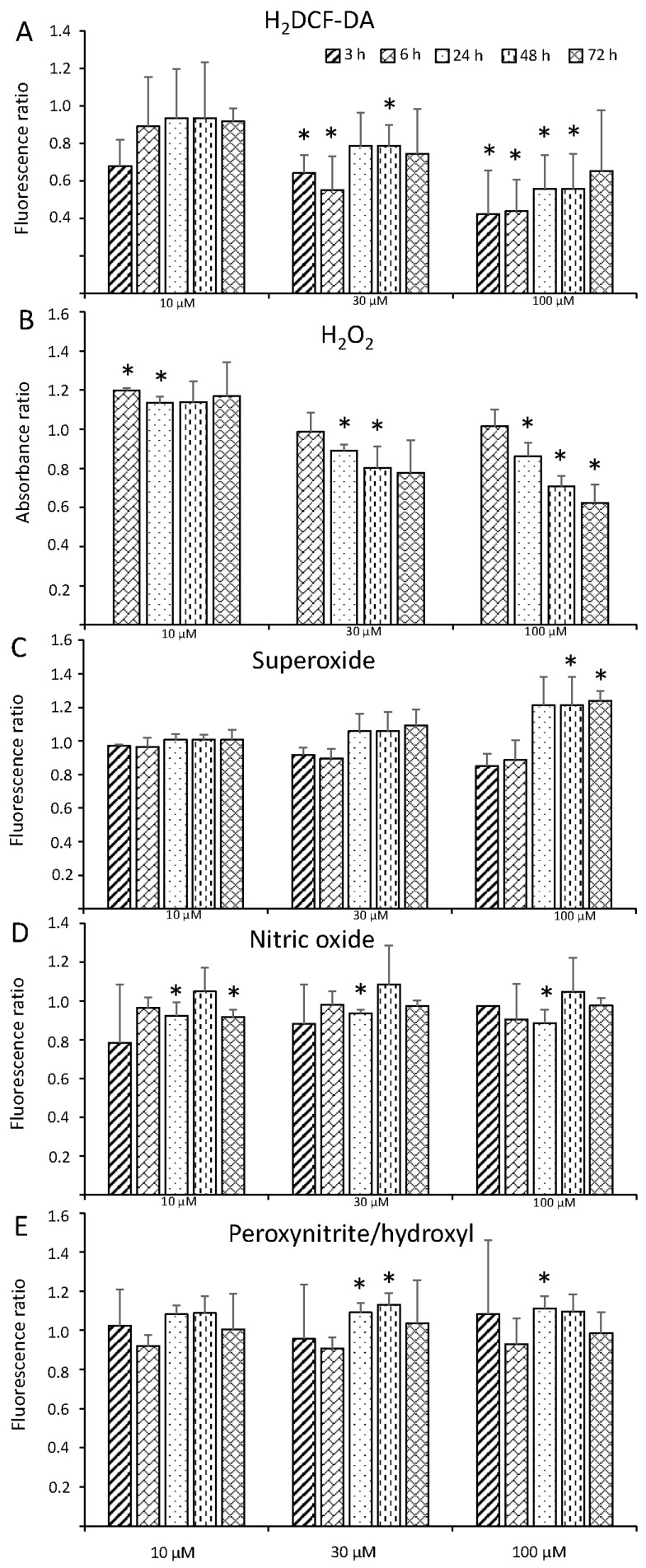
Intracellular oxidating events, as measured by H_2_DCF-DA fluorescence (**A**), extracellular H_2_O_2_ (**B**), O^2−^ (**C**), NO (**D**), and ONOO^−^/⋅OH (**E**) levels of cultures treated with 10, 30, and 100 µM HgCl_2_ for 3 h, 6 h, 24 h, 48 h, and 72 h, relative to the corresponding control values. *N* = 4 ± SD. Asterisks indicate significant differences compared to the corresponding control value, for *p* < 0.05.

Since it is well known that treatment with heavy metals, such as cadmium, often triggers ROS and RNS production, which is causally linked to toxicity [20], we wondered whether *C. velia* cultures treated with Hg^2+^ similarly experienced oxidative and nitrosative bursts. We then measured the general level of intracellular oxidative stress, as reported by H_2_DCF-DA, and quantified the production of specific ROS and RNS compounds, namely extracellular hydrogen peroxide (H_2_O_2_), superoxide (O^2−^), nitric oxide (NO), and peroxynitrite and/or hydroxyl radical (ONOO^−^/⋅OH). Being ROS and RNS signaling molecules, as well as downstream events during cell death, we extended our analysis from 3 to 72 h. Surprisingly, we observed that the intracellular oxidative environment, measured as H_2_DCF-DA fluorescence, decreased when cultures were treated with high concentrations of Hg^2+^ (30 and 100 µM) already within few hours after treatment (Figure 2A). A similar pattern was observed when extracellular H_2_O_2_ was measured (Figure 2B). H_2_O_2_ decreased at high concentrations of Hg^2+^. However, it significantly increased shortly after treatment with the lowest concentration (10 µM, 3 h, and 6 h). The decrease in H_2_DCF-DA fluorescence after exposure to high Hg^2+^ concentrations had been already observed in other studies. In particular, the oxidative burst following treatment of *Medicago sativa* root cells with 30 µM Hg^2+^ was modest and transient, occurring within the first hour of treatment [23]. At later times, H_2_DCF-DA fluorescence was markedly lower than untreated cells [22], a situation that reflects what we observed in *C. velia*. Similarly, another study observed decreased H_2_O_2_ levels 24 h after treatment of *M. sativa* roots with high (40 µM) Hg^2+^ concentration [24]. Leaves showed increased H_2_O_2_ production [24]. A different pattern in H_2_O_2_ production was also observed for two fern species under the same experimental conditions [21], suggesting that the molecular events triggered by Hg^2+^ exposure are not universal for any species and cell type.

Differently from what observed for H_2_O_2_, O^2−^ levels increased when cells were treated with high concentrations of Hg^2+^, especially 100 µM (Figure 2C). O^2−^ production appeared to be a late event since, within the first day of treatment, levels were similar to control. A late (24 h) O^2−^ induction had also been shown in *M. Sativa* leaves incubated with Hg^2+^ [24] confirming that *C. velia* responses are similar to higher plant systems.

NO production did not change dramatically after Hg^2+^ exposure, though it was significantly lower at 24 h for all treatments, and at a later time for 10 µM (Figure 2D). A decrease in NO after Hg^2+^ treatment was observed also for the microalgae *C. subellipsoidea* [26]. Interestingly, NO was produced massively in *Arabidopsis* cells treated with another heavy metal, cadmium. Moreover, NO preceded and was required for subsequent H_2_O_2_ production and programmed cell death induction [20]. The mechanism of toxicity triggered by mercury and cadmium likely follow different pathways.

Finally, we used the dye APF to quantify ONOO^−^ and/or ⋅OH, since the marker is unable to discriminate between the two reactive molecules. Also, in this case, fluorescence did not vary much among treatments, although it slightly increased at 24–48 h for 30 and 100 µM treatments (Figure 2E). ONOO^−^ forms by reaction of NO with O^2−^. Since NO levels did not vary during treatment, it was not surprising that ONOO^−^ levels were likewise stable.

Due to the limited, nonsignificant effect of treatment with Hg^2+^ at the lowest concentration (10 µM) on chlorophyll content and most of ROS/RNS production, we decided to use 30 µM as the best treatment condition for subsequent transcriptomic analyses. However, to focus on the early signaling event, and to avoid late toxicity effects, we collected cells early, 6 h after treatment.

### 3.3. Sequencing Output and Assembly

RNA samples from *C. velia* cultures treated for 6 h with 30 µM Hg^2+^ and controls were used for Illumina Genome Analyzer deep sequencing. Approximately 165 and 148 million raw reads were generated for control (Cvel_cont) and Hg^2+^-treated (Cvel_mer) cultures, respectively. After cleaning, read numbers reduced to 162 and 145 million, respectively. In total, we generated 313 million raw reads and 307 million cleans (Table 1). Among all the clean reads, more than 95% had Phred-like quality scores at the Q20 level (an error probability of 1%). The three sets of clean reads per treatment were de novo assembled into one de novo reference transcriptome with the “Trinity” program. After assembly, the six sets of clean reads were mapped to the reference transcriptome. Approximately 134 million (Cvel_cont) and 126.5 million (Cvel_mer) reads were mapped to the reference transcriptome, which accounted for 52.51% and 87.02% of the total clean reads for Cvel_cont and Cvel_mer cultures, respectively (Table 1).

### 3.4. Functional Annotation

The NCBI non-redundant (NR) database was searched using all the assembled unigenes using BLASTX tool with a cut-off E-value of 10^−5^ [68]. In addition, all unigenes were annotated by aligning with the other five public databases, including Protein family (Pfam), Cryptosporidium Genomics database (CryptoDB), a manually annotated and reviewed protein sequence database (Swiss-Prot), Protein sequence analysis and classification (InterPro), and Gene Ontology (GO) databases with a cut-off E-value of 10^−5^. Analyses showed that 52,404 and 52,388 unigenes (58.32% and 58.3% of all unigenes) were annotated in the CryptoDB and InterPro databases, respectively, while only 11,173 unigenes (12.43% of all unigenes) were annotated in the Nr database due to the absence of genome and EST information for *C. velia*. Furthermore, 16,625 unigenes (18.5% of all unigenes) were annotated in Swiss-Prot database; 3456 (3.85% of all unigenes) unigenes were annotated in the Pfam, and 1005 (1.12%) were annotated in GO databases (Table 2). Unigenes amino acid sequences were also searched for transmembrane helices using TMHMM [92] and signal peptide using SignalP v4.0 [78]. Out of total 89,853 unigenes, 1461 (1.62%) were predicted as transmembrane proteins while 1055 (1.17%) contained signal peptide pre-sequence (Table 2). Of the 89,853 unigenes, 68,713 (76.47%) returned at least one match at the E-value < 10^−5^ (Table 2).

### 3.5. Differential Expression Analysis

**Figure 3:**
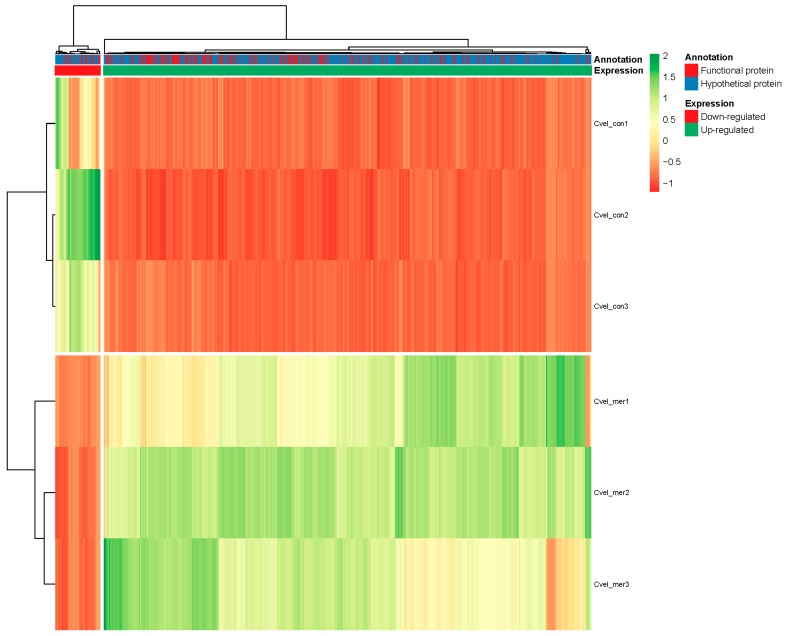
Hierarchical clustering analysis of the *C. velia* control (Cvel_cont1, Cvel_cont2, and Cvel_cont3) and Hg-treated libraries (Cvel_mer1, Cvel_mer2, and Cvel_mer3).

The read counts for unigenes were quantified based on RPKM (reads per kilobase per million) normalized matrix, to facilitate the comparison of mRNA levels. Differentially expressed genes (DEGs) (*p*-value < 0.001 and log2 (fold change) >2) were defined as genes that were significantly enriched or depleted in Hg^2+^-treated cultures compared to control (Supplementary Table S2B). Based on the six sample’s log10RPKM, hierarchical clustering of the DEGs was performed to observe the overall gene expression pattern. The green bands represent the up-regulated genes, while the red bands identify down-regulated genes (Figure 3). Two groups of DEGs with specific expression patterns were outlined from the clustering. In total, 1239 DEGs were found. Hg^2+^-treatment resulted in up-regulation of 1070 DEGs and down-regulation of only 169 DEGs (Figure 3 and Supplementary Table S2B).

**Figure 4:**
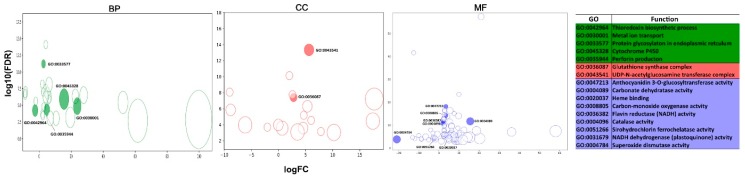
A bubble plot of the annotated differentially expressed genes (DEGs) gene expressions and gene ontology (GO) classification. The results summarized in three main categories: Biological process (BP), cellular component (CC), and molecular function (MF).

Based on sequence homology, 339 (27.36%) DEGs were annotated while the remaining 900 DEGs (72.64%) were hypothetical proteins (Figure 3 and Supplementary Table S2B). REVIOGO was used to classify and summarize the annotated DEGs (339) based on their Gene Ontology (GO) term [71]. Among these groups, the biological process (BP) category included proteins related to ‘cellular process’ (14), ‘metabolic process’ (10), and ‘single-organism process’ (4). The cellular component (CC) category comprised proteins involved in ‘cell part’ (14) and ‘organelle’ (5). Within the molecular function (MF) category, ‘binding’ (30), ‘catalytic activity’ (55), and ‘transporter activity’ (3) were represented (Figure 4).

### 3.6. Hg Exposure

#### 3.6.1. ROS-Antioxidant Defense System Related Genes

Mercury tolerance of algae and its association with the different detoxification defense systems was reported [44,93,94]. *C. velia* is one of the most remarkable endosymbiotic algae, which showed a strong ability to survive in a wide range of environmental conditions [54,95,96]. However, little is known about the molecular mechanisms of heavy metal tolerance in *C. velia*.

Based on deep sequencing of the six cDNA libraries and the GO analysis, a total of 145 ROS-related unigenes were identified (Supplementary Table S3A). Among them, 117 (80.7%) unigenes were shared by all six libraries, and 12 unigenes were specifically expressed in a single library (Cvel_cont2). Additionally, two unigenes (DN73858_c0_g1 and DN85649_c0_g1, encoding for Kynurenine 3-monooxygenase and indole-3-acetic acid amido synthetase, respectively) were only expressed in control cultures (Supplementary Table S3A). Based on the unigenes FPKM values, the expressions of 145 ROS-related genes in control and Hg^2+^-treated cultures were summarized in Supplementary Table S3A and Figure 4. Of them, 125 unigenes (86.2%) showed similar expression patterns in cultures under Hg^2+^ treatment (Cvel_mer) compared to controls (Cvel_cont), while 20 unigenes showed differential expression (DE) patterns (16 up- and four down-regulated) (Figure 4).

Many studies show that Hg^2+^ treatment modulates the production of ROS e.g., O^2−^, H_2_O_2,_ and OH [44,97,98], and ROS scavenging mechanisms, including the water–water cycle, glutathione–ascorbate (AsA-GSH) cycle, peroxidase (POD), glutathione peroxidase (GPX), and peroxiredoxin/thioredoxin (PrxR/Trx) pathways [14,99,100,101]. The water–water cycle (O^2−^–H_2_O_2_–H_2_O), mainly involves SOD, catalase peroxidase (KatG), and Prx, which sequentially convert the superoxide anion (O^2−^) to hydrogen peroxide (H_2_O_2_) and subsequently hydrogen peroxide to water [99,102]. In contrast, thioredoxins and glutaredoxins enzymes repair protein disulfides and glutathione-protein mix disulfides [103].

Superoxide dismutases (SOD) catalyze the disproportionation of O^2−^ to O_2_ and H_2_O_2_ as the first defense line of the cell against ROS [104]. Since ·O^2−^ is a precursor of several other highly reactive species, control of this free radical concentration by SOD constitutes an essential protective mechanism [105]. The *SOD* genes isoform *FeSOD* found has been reported from only a few microalgae [106]. Moreover, the activation of specific *SOD* isoforms can serve as a biomarker for cells that contains a high level of·O^2−^ due stress response [107,108]. Subsequently, the enzyme catalase (CAT) catalyzes the production of H_2_O from H_2_O_2_, while ascorbate peroxidase (APX) reduces peroxides (H_2_O_2_ and organic hydroperoxides) to H_2_O or the corresponding alcohols, respectively, using ascorbate as an electron donor [44,109]. Catalase Peroxidase (KatG) is a dual catalytic activity enzyme, acting both as catalase and as a peroxidase. Evolutionarily, KatG contains two peroxidase-like domains, an N- and a C-terminal domain. Its C-terminal domain has no known catalytic activity [110]. *KatG* probably evolved by the fusion of two copies of a rudimentary peroxidase gene (which probably also gave rise to the ascorbate and cytochrome c peroxidases) [111]. *KatG* sequences are present in the genomes of Archaea, Bacteria, and some protists [112]; its C-terminal domain has no known catalytic activity [110]. Even though it shares the same EC number with monofunctional *CAT*s, they have no sequence similarity, which suggests their different evolutionary origins [113].

Four *SOD* genes found in this study had been previously annotated in the *C. velia* genome, with two being *MnSOD*s, and two plastid *FeSOD*s (*SOD*1/2) [55] (Supplementary Table S1). Seven *V. brassicaformis SOD* loci were identified, three of them being *MnSOD*s, and four *FeSOD*s. Interestingly, we identified two plastids *KatG* in *C. velia* but none in *V. brassicaformis* nor other protists (Apicomplexa, Diplomonadida, and Parabasalidea). Possibly, their absence is compensated by other peroxidase class I enzymes (cytochrome c peroxidase (CcP) and ascorbate peroxidase (APx)) [112] (Supplementary Table S1). In this study, *FeSOD* (DN97564_c1_g1 and DN110975_c0_g1) and *MnSOD* (DN100508_c0_g1) were down-regulated by Hg^2+^ in *C. velia* (Supplementary Table S3A). Another unigene encoding *MnSOD* (DN109194_c0_g1) was unaffected by Hg. Both *FeSOD* and *MnSOD* showed lower expression levels in Hg^2+^-exposed cultures of *C. velia* than those of control cultures (Supplementary Table S3A and Figure 4). This result is in agreement with the highest ·O^2−^ production observed when cultures were treated with high concentrations of Hg^2+^ (Figure 2C). The gene for another water–water cycle enzyme, catalase-peroxidase (*katG*) (DN112103_c0_g1) was up-regulated while the other *katG* unigene (DN79540_c0_g1) was unaffected (Supplementary Table S3A, and Figure 4). Again, these results are in agreement with the observed ROS production, since H_2_O_2_ levels decrease after Hg^2+^ treatment (Supplementary Table S3A,B), probably as a result of the increased H_2_O_2_ scavenging capacity. The unigene encoding FER3 (DN112480_c0_g3) was not affected by Hg (Supplementary Table S3A). Ferritin (FER) is an iron-storage protein, has been reported to seize Fe^2+^, and to prevent the formation of ·OH [18,114,115]; its overexpression improved abiotic stress tolerance [115,116]. In our experimental system, we did not observe variation in ·OH levels following Hg^2+^ treatment (Figure 2E), a trend that correlates with the unchanged expression of its gene. Other ROS-related genes were identified in this study, although we do not have enough biochemical data to correlate their expression to ROS modulation. Glutathionylation-related enzymes (thioredoxin), encoded by unigene (DN107579_c0_g1) and other redox-related enzymes (flavin reductase (NADH), NADH dehydrogenase (plastoquinone), and kynurenine 3-monooxygenase) encoded by unigenes DN94003_c0_g1, DN99597_c0_g1, and DN7632_c0_g1, respectively, were up-regulated (Supplementary Table S3A and Figure 4).

#### 3.6.2. Xenobiotics Detoxification-Related Genes

A total of 126 of xenobiotics metabolism-related unigenes were identified in *C. velia* (Supplementary Table S3B). Only six unigenes were specifically expressed in a single library (Cvel_cont2) while all six libraries shared 120 (95.2%) unigenes. Based on the unigenes FPKM values, the expressions of 126 xenobiotics metabolism-related gene in control and Hg^2+^-treated cultures were summarized in Supplementary Table S3B and Figure 4. Of them, 111 unigenes (88.1%) did not change expression, while 15 showed different expression patterns (14 up- and only one down-regulated by Hg^2+^) (Figure 4).

Ten classes of cytochrome P450 systems (CYPs) have currently been assigned [117]. Mixed-function oxidases (MFO) class II includes most eukaryote CYPs localized to the endoplasmic reticulum and some other membranes via an N-terminal anchor and associated with a separate NADPH-cytochrome P450 reductase (P450R). The cytochrome b5 system is attributed to this activity [117,118,119]. Also, CYPs show protein–protein interactions with other CYPs to form homomeric and heteromeric complexes; these complexes can often have significant effects on CYP-mediated oxidation of substrates [120]. The analysis of the functional consequences of CYP−CYP homomeric interactions found that the complex model fits best to either a quaternary (CYP−P450R−CYP−P450R), trimeric (CYP−CYP−P450R), or dimeric (CYP−CYP) complex model, where only the binary (CYP−P450R) complex was active [120,121]. Some xenobiotic compounds are metabolized directly by phase II enzyme systems (e.g., glutathione *S*-transferases (GSTs) and UDP-glucoronyl transferases (UDPGTs) [37], while others are metabolized prior by the action of phase I enzymes. No evidence for either CYPs complexes or multixenobiotic resistance (MXR) presence exists in phytoplankton or macroalgae.

Genes encoding for biotransformation phase-I-related enzymes, including mixed-function oxidases (MFO) class II CYP (cytochrome P450 (CYP), cytochrome P450 reductase (P450R), and cytochrome b5 (Cyt b)) system, were identified in this study. One *P450R* unigene (DN11036_c0_g1) identified in our transcriptome analysis of *C. velia* (Supplementary Table S3B) had been previously annotated (Cvel_14639) [75]. Other CYP system-related enzymes were identified; 19 and 16 unigenes encoded for CYP and Cytb, respectively. Interestingly, six unigenes encoded for the binary homomeric (CYP−P450R) complex (Supplementary Table S3B). Similar to what we found in *C. velia*, the relative species *V. brassicaformis* has only one ER-P450R (Supplementary Table S1) and 19 CYP enzymes [75]. Phase-II-related enzymes (aldose reductase, glutathione S-transferase, and UDP-glucuronosyltransferase) were also identified in our study. Twenty and nine unigenes encoded for glutathione S-transferase and UDP-glucuronosyltransferase, respectively (Supplementary Table S3B). We also found two different multixenobiotic resistance (MXR) transporters (MATE and ABC transporters), phase-III-related enzymes (Supplementary Table S3B). This result is especially surprising since MXR had never been described in algae before. Chloroplastid MATE efflux protein and toxin extrusion protein were encoded by six unigenes each, while the ABC transporter was encoded by four unigenes (Supplementary Table S3B). The diversity within the ABC transporter protein family, including chromerids, has been discussed before [122], while here we report the first identification of MATE transporters in chromerids. Xenobiotics detoxification systems showed different expression patterns (Supplementary Table S3B). Genes for the phase I enzymes (CYP, Cyt b, and bifunctional P-450/NADPH-P450 reductase) were up-regulated, highlighting the importance of the CYP homomeric complex (bifunctional P-450/NADPH-P450 reductase) in *C. velia* xenobiotics metabolism; while both up-regulation (aldose reductase and UDP-glucuronosyltransferase) and down-regulation (glutathione *S*-transferase) patterns were found for phase II enzymes. *MXR* transporters-related unigenes did not change expression after Hg^2+^ treatment (Supplementary Table S3B).

#### 3.6.3. Heavy-Metal Stress Biomarkers

Fifty-seven heavy-metal stress biomarkers unigenes were identified in *C. velia* (Supplementary Table S3C). Forty-five unigenes (78.9%) were shared by all six libraries, and 12 unigenes were specifically expressed in a single library (Cvel_cont2) (Table S3C). According to the unigenes’ FPKM values, the expressions of the identified biomarkers did not change in 40 unigenes (88.9%), and only five were up-regulated (Figure 4).

Heme oxygenase (HO), glutathione synthase (GS), glyoxalase (GLY), and stress proteins (or heat shock proteins (HSPs)) are all involved in Hg^2+^ response [44,123,124,125,126]. Our transcriptomic analyses identified one unigene for chloroplastic *HO* (DN93254_c0_g1), three for *GS* and *GLY* each, and 50 for *HSP* (Table S3C). The differential expansion analysis showed that only one *GS* unigene (DN110990_c0_g1) and four *HSP70* kDa (DN113480_c11_g1, DN20133_c0_g1, DN113480_c11_g1, and DN113480_c14_g1) were up-regulated after Hg^2+^ treatment, while the rest of the unigenes were unaffected (Figure 4 and Supplementary Table S3C). The observed increase in *GS* transcript was especially interesting, since glutathione is the substrate for the synthesis of phytochelatins, the major mechanism for heavy metal chelation and detoxification in plants [127]; hence, it makes sense that *C. velia* responded to exposure to Hg^2+^ by inducing its main detoxification system.

#### 3.6.4. Phylogenetic Analyses

To provide a phylogenetic framework for interpreting our results, we constructed individual alignments for 236 *FeSOD*s and 357 *MnSOD*s proteins and concatenated these into a single alignment for phylogenetic analysis. Three *V. brassicaformis MnSOD*s (Vbra_1709, Vbra_19404, and Vbra_9701), cluster with green algae and are closely related to other stramenopiles, confirming their origin in the eukaryotic nucleus. The same applies to two sequences for *MnSOD* of *C. velia* (Cvel_4244 and Cvel_26697) (Supplementary Figure S2). The rest of the chromerid *FeSOD*s cluster within all eukaryote *FeSOD*s, suggesting their origins in a eukaryotic nucleus. *C. velia* (Cvel_3019) and *V. brassicaformis FeSOD*s sequences (Vbra_13639, Vbra_10461, and Vbra_9665) formed a sister group together with apicomplexans, while plastid *C. velia* (Cvel_7136) and mitochondrion *V. brassicaformis* (Vbra_23058) *FeSOD*s sequences formed a sister group together with kinetoplastids and amoebozoan Entamoeba dispar, respectively. *MnSOD*s and *FeSOD*s showed two disconnected branches, in agreement with previous studies [55] (Supplementary Figure S2). Phylogenetic analysis based on alignments of 138 *KatG* proteins revealed that *C. velia* had duplicated *KatG*s (Cvel_14436; Cvel_23495), originating in a eukaryotic nucleus because it branched together with stramenopiles including oomycetes, haptophyte, cryptophyte and chlorophytes (Supplementary Figure S2). This observation agrees with previous studies proposing the transfer of the KatG enzyme among eukaryotic organisms as a horizontal gene transfer (HGT) event [112,113,128].

Nineteen *CYP* genes and only one *P450R* gene were found in each chromerid (*C. velia* and *V. brassicaformis*) (Supplementary Table S2). Targeting to organelles was predicted for all these unigenes. *P450R* was predicted to be targeted to the ER in both organisms (Supplementary Table S2). They also originated in a eukaryotic nucleus, because of their position in the clade composed of all eukaryotes, forming a sister group together with apicomplexan (Supplementary Figure S2). For *CYP*s, *C. velia* had eight genes for the ER, one for the plastid, four for the cytoplasm, and six for the nucleus. *V. brassicaformis* had 10 genes for the ER, two for the plastid, one mitochondrial, four for the cytoplasm, and two for the nucleus (Supplementary Table S2). Our phylogenetic analysis showed that the ER *Chromera* (DN54179_c0_g1) and the plastid *Vitrella* (Vbra_11766) *CYP*s sequences clustered with bacteria/organelles, with planctomycetes bacteria as their closest relatives. The rest of chromerid *CYP*s sequences originated in a eukaryotic nucleus, because of their position in the clade composed of all eukaryotes (Supplementary Figure S2).

Two *HO* isoforms (*HO1* and *HO2*) were identified in *V. brassicaformis*, while the isoforms *HO*3 and *HO*4 were exclusively present in green plants [129]. *V. brassicaformis* cytoplasmic *HO2* (Vbra_20700) originated in a eukaryotic nucleus, because of the position in the clade composed of all eukaryotes, forming a sister group together with apicomplexan. The other mitochondrion *V. brassicaformis HO1* (Vbra_4541) and the only nuclear *C. velia HO1* (DN93254_c0_g1) sequences clustered with green plants and algae within the same eukaryotic clade, suggesting their origins in a eukaryotic nucleus (Supplementary Figure S2).

### 3.7. Transcription Factors in Relation to Hg Stress

The assembled transcriptome showed that 56 unique TFs were differentially expressed under Hg^2+^ stress in *C. velia* (Table 2 and Table 3). TFs could trigger the activation of the ROS-related genes expression and protect cells against oxidative damage [48,130,131,132] (Table 3). Moreover, several heavy metal-induced TFs respond to other abiotic stresses [133], suggesting that they may be crucial for the adaptation of their growth to specific environments. The TFs diversity and characteristics revealed some clues consistent with the hypothesis of endosymbioses with green and red algal symbionts in the evolutionary history of haptophytes and stramenopiles [134,135]. TF genes encoding AP2/ERF, MYB, zinc finger, and basic leucine zipper (bZIP) showed a considerable enhancement of expression in brown algae (*Ectocarpus siliculosus*) in response to heavy metal (copper) stress [136]. The regulation of specific members of TF families in *E. siliculosus* supports the hypothesis that these genes may intersect oxidative stress-related transcriptional networks [136]. Among the differentially expressed TF genes in *C. velia*, there were three classes (*NAC*, *WRKY*, and *bZIP*) involved in biological process (BP), and the rest of the TF genes were involved in cellular function process (MF) (Supplementary Table S2B).

It has been previously demonstrated that MYB TF control the expression of peroxidases and ROS homeostasis, which modulates leaf cell expansion and final organ size [137]. One *MYB* TF (DN58459_c0_g1) was differentially expressed in *C. velia,* which up-regulated under Hg-stress. This result implies that the induction or repression of *MYB* TFs may be participating in the ROS homeostasis controlled by peroxidases. Three *ERF* (DN51129_c0_g1, DN105323_c0_g1, and DN107621_c0_g1) were up-regulated; while, two *WRKY* TFs (DN75769_c0_g1 and DN103312_c0_g1) were up-regulated and one *WRKY* TFs (DN77499_c0_g1) down-regulated in *C. velia* (Supplementary Table S2A). The results suggest that the up-regulation of *WRKY* and *ERF* TFs may be involved in the mechanism of ROS scavenging for *C. velia*. *WRKY* TFs are involved in response to abiotic and biotic stresses, including heavy metals [114,138]. The overexpression of *ZmWRKY*4 in maize has elevated the expression of *SOD* and *APX* under Cd stress [48]. Moreover, the activities of SOD, POD, GST, and GPX were enhanced due to the overexpression of *ThWRKY*7 in the transgenic plant *Tamarix hispida* [131]. ERF TFs could improve plant ROS tolerance by regulating the transcription of ROS metabolic enzymes [132,139]. A recent study reported that overexpression of *TERF*1 up-regulates the transcription of *NtGPX*, which may play a vital role in the regulation of ROS scavenging under Cd stress [132].

Hg exposure significantly down-regulated some TFs, including *NAC* (DN95341_c0_g1) and *ZFP*s (DN102045_c0_g1, DN102561_c0_g1, DN105281_c0_g1, and DN27427_c0_g1) (Supplementary Table S2B). A recent study found that overexpression of *ThbZIP*1 increased the content of both soluble sugars and proteins, and it also enhanced the activity of both POD and SOD under stress conditions [47]. In *Arabidopsis*, the ZFP family were shown to positively regulate heavy metal tolerance by the regulation of the *GSH*1 expression [140]. Overexpression of the *NAC* TF family improved the osmolytes contents and reduced the H_2_O_2_ and ·O^2−^ contents under low temperature, which contribute to improving chilling stress tolerance in transgenic tobacco [141]. Overexpression of *TaNAC69-1* enhanced the expression of stress up-regulated genes such as glyoxalase protein family in transgenic wheat, suggesting that *TaNAC69* is included in regulating the drought tolerance [142]. Therefore, it seems that the changes in ROS levels observed in our study, for example the increase in ·O^2−^ levels, may ultimately depend on the modulation of TFs expression.

### 3.8. Validation of the DEGs by Real-Time RT-PCR Analysis

**Figure 5:**
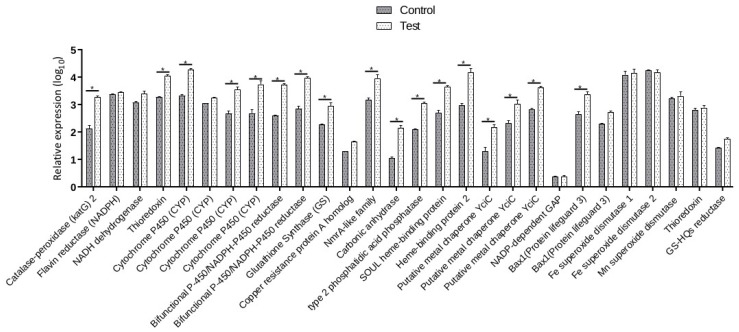
RT-qPCR analyses of 28 heavy metal stress-related genes under the control and Hg treatment in *C. velia* cultures. Each bar represents the mean ± SD of triplicate assays. Values with different letters indicate significant differences at *p* < 0.05, according to Duncan’s multiple range tests.

To evaluate the reliability and validity of our transcriptome in the identified DEGs, a total of 28 DEGs were chosen and validated by RT-qPCR analysis. As shown in Figure 5, all DEGs were differentially expressed between control cultures (Cvel_cont) and cultures under Hg^2+^ treatment (Cvel_mer). The expression patterns of Flavin reductase (*NADPH*), *katG* 2, and *GS* were significantly up-regulated by Hg exposure, and *FeSOD*1 and *FeSOD*2 were down-regulated. *CYP* and bifunctional P-450/NADPH-P450 reductase were strongly up-regulated by Hg stress in *C. velia*. Generally, RT-qPCR results were comparable with the RNA-Seq-based gene expression patterns (Supplementary Table S2B). However, *MnSOD*, thioredoxin, and Glutathionyl-hydroquinone reductase did not show consistent expression levels between RT-qPCR and Illumina sequencing data (Figure 5). The discrepancies may result from different sensitivity of the two techniques.

## 4. Conclusions

This is the first study on the responses of the alveolate alga *C. velia* to Hg^2+^ treatment. By combining physiological, biochemical, transcriptomic, and bioinformatical analyses, we were able to uncover several pathways activated in response to the heavy metal stress. In general, we observed that *C. velia* behaves like higher plant species in terms of ROS production, with a reduction in H_2_O_2_ and an increase in O^2−^ levels at high concentrations of Hg^2+^ while RNS does not seem to play a role. This is also the first transcriptomic analysis of *C. velia* altogether, an important organism for phylogenetic studies, and a model microalga. In total, 122,874 transcripts and 89,853 unigenes were successfully annotated in databases.

Moreover, the 1239 differentially expressed unigenes in *C. velia* were useful to postulate the molecular pathways involved in Hg^2+^ response, such as different detoxification defense systems and TFs. For the mechanism of ROS scavenging in response to Hg stress, the POD and glutathionylation pathways were involved, which was regulated by TFs such as ERF and WRKY. The water–water cycle and PrxR/Trx pathways might be associated with its sensitivity to Hg^2+^, which was regulated by TFs, including NAC and ZFP. Moreover, the xenobiotics detoxification system shows high activity in response to Hg stress.

Importantly, variation in expression of genes involved in ROS detoxifications generally matched with our biochemical observation of ROS levels, corroborating the validity of our dual approach. Overall, our data indicate that the response of *C. velia* to Hg^2+^ involves changes in various aspects of cell metabolism, signaling, and transport.

## Figures and Tables

**Table 1:** Summary of the sequencing results.

Sample Name	Condition	Raw Reads	Clean Reads	Total Mapped
Cvel_cont1	Control	509,407.39	499,612.65	435,130,47 (87.09%)
Cvel_cont2	Control	602,350.18	593,301.55	440,098,77 (74.18%)
Cvel_cont3	Control	539,187.31	530,589.42	464,339,73 (87.51%)
	Subtotal	165,094,488	162,350,362	133,956,897 (82.51%)
Cvel_mer1	Mercury	475,722.8	467,895.2	408,397,57 (87.28%)
Cvel_mer2	Mercury	532,104.78	523,476.04	456,650,32 (87.23%)
Cvel_mer3	Mercury	47,120.479	462,513.04	400,187,51 (86.52%)
	Subtotal	147,903,237	145,388,428	126,523,540 (87.02%)
	Total	312,997,725	307,738,790	

**Table 2:** Summary of the annotation results.

Database	Number of Unigenes	Percentage (%)
CryptoDB	52,404	58.32
InterPro	52,388	58.3
NCBI non-redundant (NR)	11,173	12.43
UniProt	16,625	18.5
Pfam	3456	3.85
GeneOntology (GO)	1005	1.12
TmHMM	1461	1.62
SignalP	1055	1.17
Annotated in at least one Database	68,713	76.47
Total Unigenes	89,853	

**Table 3:** Transcription factors identified in the *C. velia* under Hg stress.

Transcription Factors Family	Numbers
Zinc finger C_2_H_2_-type	12
Zinc finger C3H-type	9
Basic helix-loop-helix (*bHLH*)	6
Basic Leucine Zipper (*bZIP*)	5
Trihelix	5
*ERF*	3
*WRKY*	3
B3	3
GATA	3
*NAC*	3
ADP-ribosylation factor (*ARF*)	2
Homeodomain-leucine zipper (*HD-ZIP*)	1
*MYB*	1
Total	56

## Supplementary Materials

The following are available online at https://www.mdpi.com/2218-273X/9/11/647/s1. **Figure S1.** Growth curve of *C. velia* culture. *N* = 3 ± SD. Arrow indicates the time of treatment (10 days after subculturing). **Figure S2.** Phylogenetic supertrees of all selected detoxification-related enzymes (heme oxygenase (*HO*), superoxide dismutase (*SOD*), NADPH-cytochrome P450 reductase (*P450R*), Cytochrome P450 (*CYP*) and Catalase-peroxidase (*katG*)), which explores the evolutionary relationships between *C. velia* enzymes and other domains of life based on protein sequences. **Table S1.** Summary of the protein sub-cellular localization of the identified detoxification-related enzymes (heme oxygenase (HO), superoxide dismutase (SOD), NADPH-cytochrome P450 reductase (P450R), Cytochrome P450 (CYP) and Catalase-peroxidase (katG)) in the *C. velia* and its close relative species (*Vitrella brassicaformis*). **Table S2.** (A) Primers for qRT-PCR analysis and (B) differentially expressed genes in *C. velia*. **Table S3.** Gene expression of selected (A) ROS-antioxidant defense system-related genes, (B) Xenobiotics detoxification-related genes and (C) Heavy metal-stress biomarkers in response to Hg exposure in *C. velia*.
