# Supplementary figures and images for: Transcriptomic Analysis Reveals the Roles of Detoxification Systems in Response to Mercury in Chromera velia
Source: Biomolecules. 2019 Oct 24;9(11):647. doi: 10.3390/biom9110647 (PMC6920818; doi:10.3390/biom9110647)

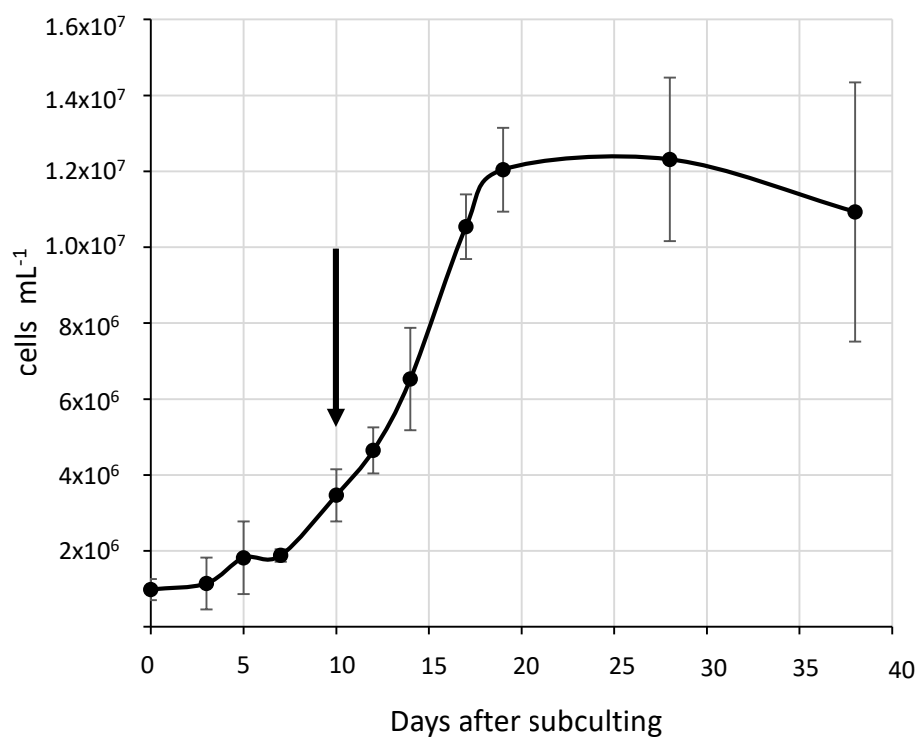

Supplement: Supplementary file 1 [file biomolecules-09-00647-s001.zip › supplementary files/Figure S1.pdf]

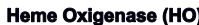

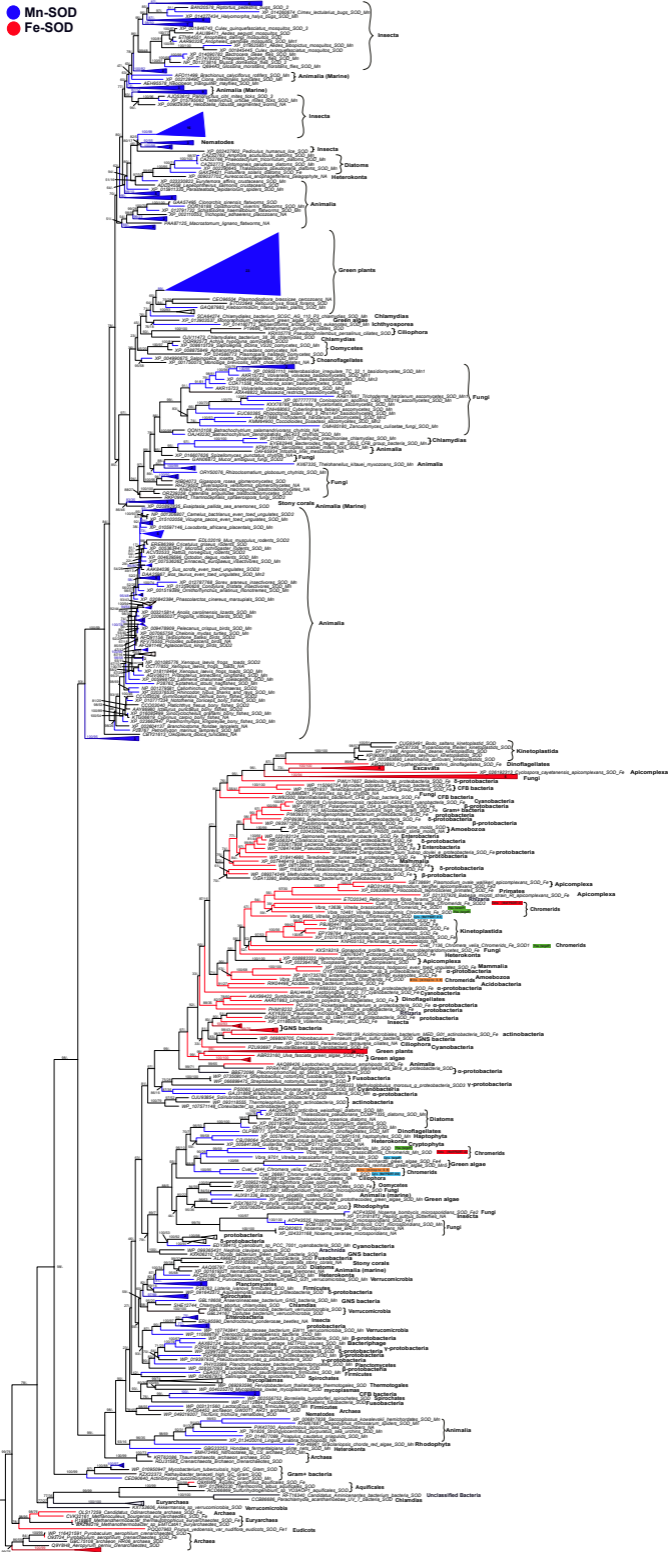

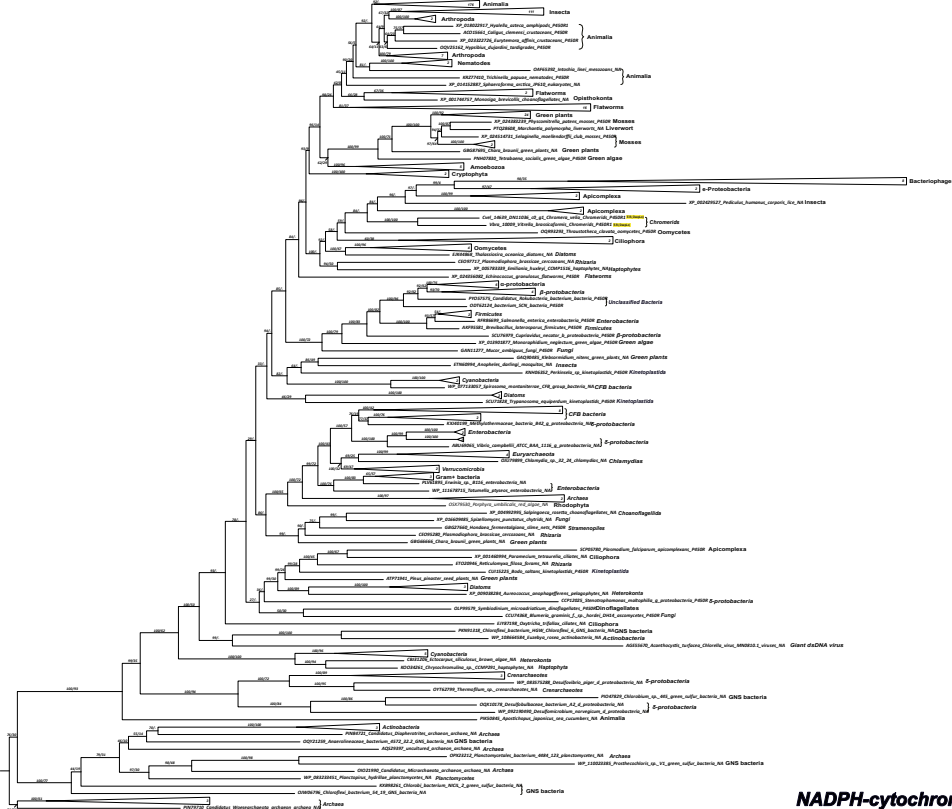

**NADPH-cytochrome P450 reductase (P450R)**

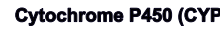

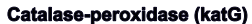

Supplement: Supplementary file 1 [file biomolecules-09-00647-s001.zip › supplementary files/Figure S2.pdf]
